# Adverse events following immunization against SARS-CoV-2 (covid-19) in the state of Minas Gerais

**DOI:** 10.11606/s1518-8787.2021055003734

**Published:** 2021-10-05

**Authors:** Roberta Barros da Silva, Thales Philipe Rodrigues da Silva, Ana Paula Sayuri Sato, Francisco Carlos Felix Lana, Josianne Dias Gusmão, Janaina Fonseca Almeida Souza, Fernanda Penido Matozinhos

**Affiliations:** I Secretaria de Estado da Saúde de Minas Gerais Belo HorizonteMinas Gerais Brasil Secretaria de Estado da Saúde de Minas Gerais. Belo Horizonte, Minas Gerais, Brasil; II Universidade Federal de Minas Gerais Faculdade de Medicina Programa de Pós-Graduação em Ciências da Saúde – Saúde da Criança e do Adolescente Belo HorizonteMinas Gerais Brasil Universidade Federal de Minas Gerais. Faculdade de Medicina. Programa de Pós-Graduação em Ciências da Saúde – Saúde da Criança e do Adolescente. Belo Horizonte, Minas Gerais, Brasil; III Universidade de São Paulo Faculdade de Saúde Pública Departamento de Epidemiologia São PauloSP Brasil Universidade de São Paulo. Faculdade de Saúde Pública. Departamento de Epidemiologia. São Paulo, SP, Brasil; IV Universidade Federal de Minas Gerais Escola de Enfermagem Departamento de Enfermagem Materno-Infantil e Saúde Pública Belo HorizonteMinas Gerais Brasil Universidade Federal de Minas Gerais. Escola de Enfermagem. Departamento de Enfermagem Materno-Infantil e Saúde Pública. Belo Horizonte, Minas Gerais, Brasil

**Keywords:** Coronavirus Infections, prevention & control, Vaccines, adverse effects, Clinical Trials Data Monitoring Committees

## Abstract

**OBJECTIVE:**

To analyze adverse events following immunization (AEFI) against SARS-CoV-2 (covid-19) in the state of Minas Gerais (MG), Brazil.

**METHODS:**

Epidemiological, descriptive study, with data from e-SUS *Notifica* (e-SUS Notification) in the state of Minas Gerais from January 20 to March 5, 2021. All suspected cases of AEFI of the covid-19 vaccine in the state were analyzed, totaling 7,305 cases. In this study, we verified the possible correlation between AEFI and the possible immunobiological administered causalities. The variables analyzed for AEFI cases were the immunobiological agent administered (AstraZeneca or Coronavac), the type of event, the evolution of the case, and the time in days since the administration of the immunobiological agent and the onset of symptoms and causality. The incidence rate (IT) was calculated for 100,000 doses applied.

**RESULTS:**

The occurrence of AEFI as a result of the covid-19 vaccine was frequent (TI: 777.12) in the state. However, only 3% were classified as a severe AEFI, with a 20.85 IT, and 4.71% of them evolved to deaths (8.19 deaths per 100,000 doses applied). Among the deaths analyzed, 84.4% were classified as preexisting conditions caused by factors other than vaccines. Regarding non-serious AEFI, 1.11% occurred by immunization errors (TI: 8.62 EI for every 100 thousand doses applied).

**CONCLUSION:**

This work encourages the discussion about the importance of recording AEFI related to covid-19 vaccines, demonstrating its safety for the population.

## INTRODUCTION

Since November 2019, the world has suffered the consequences and transformations caused by a new virus, called SARS-CoV-2, and the related disease, the covid-19^1^. Nowadays we live a pandemic^2,3^ or syndemic^4^ situation given its rapid spread^1,5^ and the occurrence of new variants^6^.

The spread of this virus has been rapid in several countries^1,5^. Until April 6, 2021, 131,593,180 cases of covid-19 were confirmed worldwide, with 2,856,632 deaths, distributed in 200 countries^7^. In Brazil, 13,013,601 cases and 332,752 deaths have been confirmed^8^. A study with 250,000 cases of covid-19 in Brazil showed a wide distribution of the disease in all regions of the country, resulting in a high overall burden of the disease^9^. In this epidemiological context, a new covid-19 vaccine will need to cover at least 55% of the population to provide collective immunity, reaching 85% depending on the country^10^.

The transmission of SARS-CoV-2 can occur by droplets, by contact, or by aerosol. The first occurs by the ingestion or inhalation of droplets expelled by an infected person when coughing or sneezing. The second occurs when an individual touches a surface or contaminated object. The third form of contamination is the indoor contact^2,3^. From a clinical point of view, the infection presents with a febrile condition associated with respiratory symptoms with cough, which can progress to bilateral pneumonia. Severe cases of the disease are usually in older patients and people with comorbidities, such as hypertension, diabetes, and cardiovascular diseases^11^.

A worldwide effort to develop a vaccine against this virus began, in view of the seriousness of the disease, the high rate of transmission, and the high demand for health service by infected patients. Several technologies are used to produce vaccines against SARS-CoV-2 and at a surprising speed. In less than 6 months, different vaccine candidates have reached the clinical stage. In Brazil, given the epidemiological emergency resulting from covid-19, the country established a temporary authorization for the emergency use of covid-19 vaccines on an experimental basis to face a public health emergency^12^.

The *Agência Nacional de Vigilância Sanitária* (Anvisa - National Health Surveillance Agency), the Brazilian State’s regulatory body, authorized Coronavac vaccines, an inactivated immunizing agent, developed by the Chinese laboratory *Sinovac Life Sciences Co. Ltd*, in partnership with the *Instituto Butantan* (IB - Butantan Institute), of the State of São Paulo, Brazil, and the non-replicating viral vector vaccine ChAdOx1 nCoV-19, developed by Oxford University and pharmaceutical company AstraZeneca, with technology transfer to *Fundação Oswaldo Cruz* (Fiocruz - Oswaldo Cruz Foundation) – Institute of Technology in Immunobiologicals – Bio-Manguinhos and the Indian laboratory *Serum Institute of India Pvt. Ltd*^12^.

In a scenario of the introduction of a recent vaccine to the population, the pharmacovigilance of Adverse Events Following Immunization (AEFI) is extremely relevant^12,13^. Any serious, undesirable or unexpected sign or symptom manifested in an individual who has received any type of immunobiological is considered an AEFI and can be caused by several factors related to the components of the immunobiological, the vaccination process, or the person already vaccinated^14,15^.

The Ministry of Health (MH) establishes that all AEFI, related with the definitions of cases already established in the Manual de *Vigilância Epidemiológica de Eventos Adversos Pós-Vacinação* (Manual for Epidemiological Surveillance of Adverse Post-Vaccination Events), must be notified^15^, according to the *Programa Nacional de Imunização* (PNI - National Immunization Program) and the e-SUS *Notifica* system^12,15,16^.

AEFI can be classified as a Serious Adverse Events (SAE), which is an event that requires hospitalization, compromises the patient, that is, that causes risk of death and that requires immediate clinical intervention to prevent death, causes significant dysfunction and/or permanent disability, results in congenital anomaly or causes death; or a Non-Serious Adverse Events (NSAE), which are all those events that do not meet the SAE criteria^15^. Immunization errors (IE) are adverse events caused by inadequate handling, prescriptions and/or administration and are preventable by personnel training, adequate supply of equipment and supplies for vaccination, and supervision of services^15^.

Given the recent introduction of anti-covid-19 vaccines, trust in any of these immunobiological will be crucial forsuccess^17^ and AEFI can contribute to the lack of confidence, decrease in adherence to the vaccine schedule, and consequently in vaccination coverage. Besides, AEFI could increase the situation of vulnerability of the population in relation to vaccine-preventable diseases^18^. Thus, pharmacovigilance studies are very important to better understand the scenario. So far, a systematic review shows that AEFI caused by vaccines against covid-19 were resolved within 24 hours after vaccination, with a more local adverse reaction. Pain or sensitivity in the region of application is common, in addition to fatigue, fever, or bodypain^19^. We intend to analyze the AEFI against SARS-CoV-2 (COVID-19) in the state of Minas Gerais (MG), Brazil.

## METHODS

**Figure 1:**
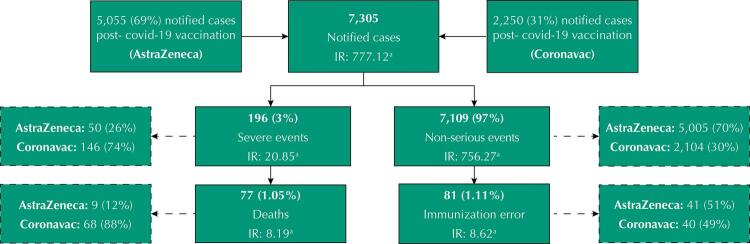
Suspected cases of AEFI-covid-19 notified in the e-SUS Notification information system, from January 20 to March 5, 2021. Minas Gerais, Brazil.

^a^ AEFI Incidence rate for 100,000 doses applied.

This is an epidemiological, descriptive study, based on data from e-SUS *Notifica* (e-SUS Notification) in the state of Minas Gerais from January 20 to March 5, 2021. All suspected cases of AEFI of the covid-19 vaccine were analyzed, totaling 7,305 cases. The sample selection flowchart for AEFI can be seen in Figure 1.

The state of MG has 853 municipalities, in a 586,522,122 km^2^territorial range and an estimated population of 21,168,791 inhabitants in 2019^20,21^. For the organization and planning of health care (given its demographic, socioeconomic, geographic, sanitary, epidemiological, service provision, and relations between municipalities), the state is divided into 14 health macro-regions: South; South Center; Center; Jequitinhonha; West; East; Southeast; North; Northwest; Southeast; North East; Southern Triangle; Northern Triangle and Steel Valley.

For this study, we analyzed suspected cases of AEFI with and without closure. The variables analyzed for AEFI cases were the immunobiological administered (AstraZeneca or Coronavac); the type of event (non-serious, severe, immunization error or immunization error with adverse event); the evolution of the case (cure without sequelae, cure with sequelae, in follow-up, it is not AEFI, death or others); and the time in days between the administration of the immunobiological agent and the onset of symptoms. The classification of AEFI causality was also considered (consistent temporal relationship, but without evidence in the literature to establish a causal relationship, the investigation data are conflicting in relation to causality, unclassifiable and inconsistent, or coincident association)^15^. The Incidence Rate (IT) was also calculated for 100,000 doses applied according to the following formula:

$$IT=\left(\frac{AEFI}{\text{ Number of doses applied }}\right)\times 100.000\text{ doses applied }$$

The numerator considered the total number of AEFI cases of the covid-19 vaccine; we used the doses of the same vaccine administered in the period as the denominator. We obtained the number of doses from the website of the *Secretária Estadual de Saúde do Estado de Minas Gerais* (SES - MG - State Health Department of the State of Minas Gerais), information collected on March 5, 2021, being the deadline which comprises this study the period between January 20 and March 5, 2021.

For data analysis, we used the *Statistical Software for Professional* (Stata) program, version 16.0. Estimates of AEFI were presented in proportions (%), according to the immunobiological agent administered (AstraZeneca or Coronavac), age group, and sex, in addition to the type of event and case evolution. For the interval between vaccine administration and the onset symptoms (in days), we presented the data as median, because of the lack of normality in the distribution of the variable assessed by the Shapiro-Wilk test.

The research was approved by the Ethics Committee of the Universidade Federal de Minas Gerais, protocol CAAE 53843716.0.0000.5149.

## RESULTS

Between January 20 and March 5, 2021, 940,013 doses were administered in Minas Gerais, most of them as a first dose (633,032) and 306,981 as a second shot. In the same period, 7,305 cases of AEFI were reported, corresponding to 0.45% of the total administered doses, with an incidence rate of 777.12 cases per 100,000 doses applied. Most cases (69%) were due to immunobiological tests from the AstraZeneca laboratory (Figure 1).

According to the AEFI classification, 3% were considered an SAE (IT: 20.85 AEFI– EAG for every 100 thousand doses applied), and 4.71% of these events evolved into deaths (IT: 8.19 deaths in every 100 thousand doses applied). However, 97% of the notified cases were classified as NSAE and 1.11% were EI (IT: 8.62 IE for every 100 thousand doses applied) (Figure 1).

Seven (0.09) of the IE cases were also associated with adverse events (AE), with an IT of 0.74 per 100,000 doses.

Considering the total number of notifications, 43.6% affected people aged between 18 and 35 years, with more incidence on women (83.4%). The time between vaccination and onset of symptoms had a median of six days (Table 1).

**Table 1:** Distribution of suspected AEFI cases – covid-19, by age group, sex and time between vaccination and onset of symptoms. Minas Gerais, Brazil. 2020.

Variables	n	%
Age group (years)		
13 to 17 years	4	0.1
18 to 35	3,182	43.6
36 to 49	2,736	37.5
50 to 64	969	13.3
≥ 65	398	5.4
Not provided	16	0.2
Sex		
Male	1,204	16.5
Female	6,101	83.5
Vaccination and onset of symptoms (days)^a^	6 (0–44)	

^a^ Median (minimum and maximum).

Regarding the SAE, the majority (60.7%) affected people aged 65 years or more and females (65.3%). Symptoms started eight days after vaccine administration (Table 2).

**Table 2:** Distribution of suspected cases of SAE - covid-19, by age group, sex and time between vaccination and onset of symptoms. Minas Gerais, Brazil. 2020.

Variables	n	%
Age group (years)		
18 to 35	25	12.8
36 to 49	28	14.3
50 to 64	24	12.2
≥ 65	119	60.7
Sex		
Male	68	34.7
Female	128	65.3
Vaccination and onset of symptoms (days)^a^	8 (0–35)	

^a^ Median (minimum and maximum).

77 deaths were reported and the majority (89.6%) were people aged 65 years or older and female (57.1%). The onset of symptoms occurred eight days after the administration of the vaccine; 84.4% were classified as having pre-existing conditions, caused by factors other than the vaccine (Table 3).

**Table 3:** Distribution of deaths – covid-19, by age group, sex, time between vaccination and onset of symptoms and classification of causality. Minas Gerais, Brazil. 2020.

Variables	n	%
Age group (years)		
18 to 35	1	1.3
36 to 49	3	3.9
50 to 64	4	5.2
≥ 65	69	89.6
Sex		
Male	33	42.9
Female	44	57.1
Vaccination and onset of symptoms (days)^a^	8 (0 – 35)	
Classification according to causality		
Consistent temporal relationship, but no evidence in the literature to establish a causal relationship	1	1,3
Preexisting conditions caused by factors other than vaccines	65	84,4
Under investigation.	11	14,3

^a^ Median (minimum and maximum).

Most deaths were residents of long-term care facilities for the elderly and with comorbidities such as adenocarcinoma of the prostate, chronic obstructive pulmonary disease, malignant prostate cancer, diabetes, congestive heart failure, hypertension, and end-stage renal disease. The death diagnoses characterized them as septicemia, cardiac arrest, stroke, acute myocardial infarction, congestive heart failure, bacterial infection, urinary tract infection, and hypertension secondary to endocrine disorders.

In the reported cases of immunization errors, 43.2% occurred to people aged between 18 and 35 years, mostly women (80.2%) and, of the total, seven (8.6%) were IE with AE (TI: 0.74 for every 100 thousand doses applied).

**Figure 2:**
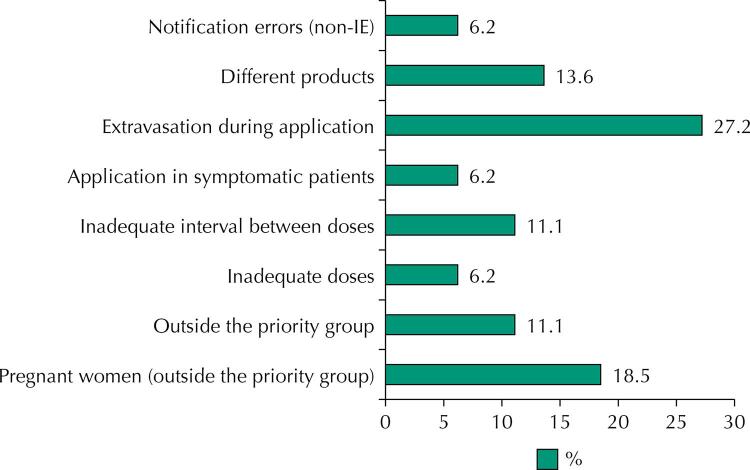
Classification of immunization errors according to the type of occurrence (n = 81).

The classification of IE, according to the type of occurrence, can be seen in Figure 2. The majority (27.2%) of IE were due to extravasation, followed by vaccination in pregnant women (18.5%) outside the priority group (Figure 2).

## DISCUSSION

This study demonstrates that the AEFI is a frequent result of the vaccine against covid-19 in Minas Gerais. However, only 3% were classified as severe, with an IT of 20.85 per 100,000 doses applied, and 4.71% of the SAE evolved to death (IT: 8.19 deaths per 100,000 doses applied). Among the deaths analyzed, most of them (84.4%) were classified as caused by pre-existing conditions of other factors and not by vaccines, demonstrating that so far none of the deaths had a causal relationship with covid-19 vaccines. Regarding EANG, 1.11% were due to IE (IT: 8.62 IE for every 100 thousand doses applied)

Our results are aligned with data published in the Boletim Epidemiológico do Ministério da Saúde (Epidemiological Bulletin of the Ministry of Health - 2021), in which the majority of notified cases were classified as NSAE and affected females with a higher incidence. Regarding SAE and deaths, both IT were lower than the national incidence rate for the same event^21^ (IT - SAE: 0.21 *versus* 7.1 per 1,000 doses applied; IT - death: 0.08 *versus* 2,4 per 1,000 doses applied, the rates of these data were transformed to 1,000 doses, for comparison with the Bulletin)^22^. The data reinforce the safety and efficacy of anti-covid-19 immunobiological available in Brazil^23,24^.

The sociodemographic profile data of the cases show a higher incidence of AEFI in women aged between 18 and 35 years, results that were already expected, due to the flow of priority groups followed by the vaccination scheme against covid-19, starting with health professionals^12^, most of them are nursing professionals, in which there is a predominance of women (85.1%)^25^, leading to the fact that by March 5, 2021, 442,099 health professionals received the first dose of the vaccine and another 246,565 had already completed the vaccine scheme against covid-19^26^.

Most deaths occurred among residents in Skilled Nursing Facilities (SNF). They were patients extremely vulnerable, debilitated, bedridden, or with walking difficulties, in addition to presenting care involving dialysis and with multiple comorbidities. The older adults, especially residents in a SNF, are also classified as a priority group for vaccination against covid-19 in Brazil^12^.

The *Global Advisory Committee on Vaccine Safety* (GACVS) subcommittee for the covid-19 vaccine safety at a meeting on January 19, 2021, reviewed reports of deaths in frail older adults vaccinated with the Pfizer-BioNTech COVID-19 dose (vaccine not yet used in Brazil during the study) and concluded that “current reports do not suggest any unexpected or unfavorable increase in fatalities in frail older adults or any unusual features of adverse events after administration of Pfizer-BioNTech COVID-19 vaccine”^27^.

In Brazil, the Information Note No. 11/2021-CGPNI/DEIDT/SVS/MS, of February 2, 2021, was emitted in order to clarify the adverse events supposedly attributable to vaccination against covid-19. It concludes that it cannot be affirmed the existence of an AEFI with a proven causal association and it reinforces that, of the 37,765 doses applied to older residents in SNF, up to the date of the informative note, the incidence of deaths among them was 34.4 per 100,000 doses applied, a rate considered lower than the baseline mortality rate observed in this population in Brazil (325 to 916 deaths per month per 100,000 older adults living in SNF)^28^.

The occurrence of immunization errors was low and, in particular, the number of IE with AE had a 0.74 IT per 100,000 doses, which can be considered as any preventable event that could cause or lead to inappropriate use of immunobiological or cause harm to the patient^13^. IE can be classified as production error (non-compliance with good manufacturing practices that can lead to quality deviation, such as potency changes and increased reactogenicity); error in the cold chain (vaccine transported or stored incorrectly); error in handling; and administration error (non-sterile injection, reconstitution error, injection in the wrong place, ignored contraindication, expired vaccine), which occur due to non-compliance with standards and techniques, which may result in an adverse event^29,30^.

The IE that resulted in adverse events in this study, presented IT less than 1 per 100,000 doses applied. As preventable events, it is necessary that immunization coordinations reinforce training on the correct vaccination technique^31^, especially in municipal ones and where there is emergency hiring of vaccinators. However, in mass vaccination campaigns, with immunization against covid-19, it is expected that immunization errors occur^31^.

The most common IEs were extravasation and administration in pregnant women outside the priority group. According to Information Note No. 21/2021-CGPNI/DEIDT/SVS/MS, of March 3, 2021, the occurrence of extravasation, whether during or after application, at the injection site or at the syringe-needle connection, should be considered as EI and, consequently, notified^31^. However, the Note reinforces that it is unlikely that extravasation of small volumes will cause a worse immune response on the part of the vaccinated person and, consequently, extra doses are not recommended^31^.

A preliminary cohort study with a small number of pregnant participants, evaluating serum and breast milk, demonstrated that covid-19 mRNA vaccines (Pfizer or Moderna, not yet linked by the Brazilian Ministry of Health, at the time of the research, to vaccinate the population), generated a satisfactory humoral response in pregnant and lactating women, with immunogenicity and reactogenicity similar to those observed in non-pregnantwomen^32^.

Technical Note No. 1/2021-DAPES/SAPS//MS, of March 11, 2021, reinforces that so far there is no evidence to contraindicate the vaccines available in Brazil for pregnant, postpartum, and breastfeeding women. Moreover, the document adds that those women belonging to the priority groups must be immunized^33^. In Brazil, the Brazilian Covid-19 and Pregnancy Study Group identified that comorbidities are conditions significantly associated with mortality in the population of pregnant women^34^.

This study, however, has some limitations, as it is developed based on data from secondary databases, limited to specific information present in the notification form, which may have inconsistencies because the insertion of notifications in e-SUS *Notifica* is performed by several professionals. Furthermore, 14.3% of deaths in the period are still under investigation. However, taking into account the percentages of the causes of deaths investigated, we assumed that there would be no change in the conclusion of this study. All these factors make the closure and causality attribution process slower, thus slowing down the analysis of the data produced. The *Unidades Regionais de Saúde* (Regional Health Units) are frequently required to present such inconsistencies in an attempt to improve the process with the notifying municipalities, improving the quality of the data produced by the central level (technical area of AEFI of the *Coordenação de Imunização da Secretaria Estadual de Saúde de Minas Gerais*/ Coordination of Immunization of the State Secretariat of Minas Gerais Health).

## CONCLUSION

Most of the suspected AEFI cases were considered NSAE. Therefore, this work encourages the discussion on the importance of recording AEFI resulting from vaccines against covid-19, demonstrating that they are safe for the population.

The results found also suggest that post-covid-19 vaccine deaths were not related to vaccination, but to the preexisting conditions caused by factors other than vaccines.
