# Supplementary material for: Adverse events following immunization against SARS-CoV-2 (covid-19) in the state of Minas Gerais
Source: Rev Saude Publica. 2021 Oct 5;55:66. doi: 10.11606/s1518-8787.2021055003734 (PMC8522717; doi:10.11606/s1518-8787.2021055003734)
Supplement: Translation

# Translation: Eventos adversos pós-vacinação contra o SARS-CoV-2 (covid-19) no estado de Minas Gerais

**Authors:** Roberta Barros da Silva, Thales Philipe Rodrigues da Silva, Ana Paula Sayuri Sato, Francisco Carlos Felix Lana, Josianne Dias Gusmão, Janaina Fonseca Almeida Souza, Fernanda Penido Matozinhos

**Affiliations:** I Brasil Secretaria de Estado da Saúde de Minas Gerais. Belo Horizonte, MG, Brasil; II Brasil Universidade Federal de Minas Gerais. Faculdade de Medicina. Programa de Pós-Graduação em Ciências da Saúde – Saúde da Criança e do Adolescente. Belo Horizonte, Minas Gerais, Brasil; III Brasil Universidade de São Paulo. Faculdade de Saúde Pública. Departamento de Epidemiologia. São Paulo, SP, Brasil; IV Brasil Universidade Federal de Minas Gerais. Escola de Enfermagem. Departamento de Enfermagem Materno-Infantil e Saúde Pública. Belo Horizonte, Minas Gerais, Brasil

**Keywords:** Infecções por Coronavirus, prevenção & controle, Vacinas, efeitos adversos, Comitês de Monitoramento de Dados de Ensaios Clínicos

## Abstract

**OBJETIVO:**

Analisar os eventos adversos pós-vacinação (EAPV) contra o SARS-CoV-2 (covid-19) no estado de Minas Gerais (MG).

**MÉTODOS:**

Estudo epidemiológico, descritivo, com dados do e-SUS Notifica no estado de Minas Gerais durante o período de 20 de janeiro a 5 de março de 2021. Foram analisados todos os casos suspeitos de EAPV da vacina contra covid-19 no estado, totalizando 7.305 casos. Para este estudo, verificou-se a possível correlação entre os EAPV e a causalidade com o imunobiológico administrado no período estabelecido. As variáveis analisadas para os casos de EAPV foram o imunobiológico administrado (AstraZeneca ou Coronavac), o tipo de evento, a evolução do caso e o tempo em dias da administração do imunobiológico e o início dos sintomas e causalidade. Calculou-se a taxa de incidência (TI) para 100 mil doses aplicadas.

**RESULTADOS:**

A ocorrência dos EAPV em decorrência da vacina contra covid-19 foi frequente (TI: 777,12) no estado. Entretanto, somente 3% foram classificados como EAPV grave, com TI de 20,85, sendo que 4,71% deles evoluíram para óbitos com TI (8,19 óbitos a cada 100 mil doses aplicadas). Dentre os óbitos analisados, 84,4% foram classificados como condições preexistentes causadas por outros fatores e não por vacinas. Em relação aos EAPV não graves, 1,11% foram em decorrência de erros de imunização (TI: 8,62 EI a cada 100 mil doses aplicadas).

**CONCLUSÃO:**

Esse trabalho fomenta, portanto, a discussão sobre a importância dos registros dos EAPV decorrentes das vacinas contra covid-19, demonstrando sua segurança para a população.

## INTRODUÇÃO

Desde novembro de 2019, o mundo sofre as consequências e transformações causadas por um novo vírus, denominado SARS-CoV-2, e a doença que desenvolve, a covid-19^1^, hoje reconhecida como pandemia^2,3^ ou uma sindemia^4^, devido à rápida disseminação^1,5^ e as novas variantes^6^.

A propagação desse vírus tem se mostrado rápida em diversos países^1,5^, até 6 de abril de 2021, 131.593.180 casos de covid-19 foram confirmados em todo o mundo, com 2.856.632 mortes, distribuídas em 200 países^7^. No Brasil, já foram confirmados 13.013.601 casos e 332.752 óbitos^8^. Estudo com 250.000 casos de covid-19 no Brasil evidenciou ampla distribuição da doença em todas as regiões do país, resultando em uma alta carga geral da doença^9^. Nesse contexto epidemiológico, uma nova vacina covid-19 precisará ter sua cobertura vacinal de pelo menos 55% da população para fornecer imunidade coletiva, chegando a 85% dependendo do país^10^.

A transmissão do SARS-CoV-2 pode ocorrer por gotículas, por contato ou ainda por aerossol: a primeira ocorre a partir da ingestão ou inalação de gotículas expelidas por pessoa contaminada ao tossir ou espirrar, a segunda acontece quando um indivíduo toca uma superfície ou objeto contaminado., e a terceira forma de contaminação é por estar em ambientes fechados^2,3^. Do ponto de vista clínico, a infecção se apresenta com quadro febril associado a sintomas respiratórios com tosse e que pode evoluir para pneumonia bilateral. Sabe-se que os casos graves da doença, geralmente, são em pacientes com idade mais avançada e pessoas com comorbidades, como hipertensão, diabetes e doença cardiovascular^11^.

Diante da gravidade da doença, da alta taxa de transmissibilidade e da alta demanda do serviço de saúde por parte dos pacientes infectados, iniciou-se uma busca em todo o mundo pelo desenvolvimento de uma vacina contra esse vírus. Várias tecnologias são utilizadas para produção das vacinas contra o SARS-CoV-2 e em velocidade surpreendente, sendo que, em menos de 6 meses, diferentes candidatos vacinais chegaram à fase clínica. No Brasil, diante do quadro de emergência epidemiológica decorrente da covid-19, estabeleceu-se a autorização temporária de uso emergencial, em caráter experimental, de vacinas contra a covid-19 para o enfrentamento da emergência de saúde pública de importância nacional^12^.

A Agência Nacional de Vigilância Sanitária (Anvisa), como órgão regulador do Estado brasileiro, concedeu a autorização às vacinas Coronavac, que é um imunizante inativado, desenvolvida pelo laboratório chinês *Sinovac Life Sciences Co. Ltd*, em parceria com o Instituto Butantan (IB), do Estado de São Paulo, Brasil, e a vacina de vetor viral não replicante ChAdOx1 nCoV-19, desenvolvida pela Universidade de Oxford e a farmacêutica AstraZeneca, com transferência de tecnologia para a produção da vacina pela Fundação Oswaldo Cruz (Fiocruz) – Instituto de Tecnologia em Imunobiológicos – Bio-Manguinhos e com o laboratório indiano *Serum Institute of India Pvt. Ltd*^12^.

Em um cenário de introdução de uma vacina recente para a população, a farmacovigilância dos Eventos Adversos Pós-Vacinação (EAPV) tem extrema relevância^12,13^. Qualquer sinal ou sintoma grave, indesejável ou inesperado, manifestado no indivíduo que tenha recebido qualquer tipo de imunobiológico é considerado um EAPV e pode ser causado por vários fatores relacionados aos componentes do imunobiológico, ao processo de vacinação ou ao vacinado^14,15^.

O Ministério da Saúde (MS) estabelece que todos os EAPV, em consonância com as definições de casos já estabelecidos no Manual de Vigilância Epidemiológica de Eventos Adversos Pós-Vacinação, deverão ser notificados^15^, processo que segue o fluxo estabelecido pelo Programa Nacional de Imunização (PNI), por meio do sistema e-SUS Notifica^12,15,16^.

Os EAPV podem ser classificados, como: Eventos Adversos Graves (EAG), qualquer evento que requeira hospitalização, comprometa o paciente, ou seja, que ocasione risco de morte e que exija intervenção clínica imediata para evitar o óbito, cause disfunção significativa e/ou incapacidade permanente, resulte em anomalia congênita ou ocasione o óbito; Eventos Adversos Não Graves (EANG), todos aqueles que não se enquadram nos critérios de EAG^15^. Os erros de imunização (EI) são eventos adversos causados por manuseio, prescrições e/ou administração inadequados e são preveníeis por meio de treinamento de pessoal, de suprimento adequado de equipamentos e insumos para a vacinação e da supervisão dos serviços^15^.

Diante da introdução recente das vacinas anticovid-19, a confiança em qualquer desses imunobiológicos será crucial para seu sucesso^17^ e os EAPV podem contribuir para essa falta de confiança, diminuição na adesão ao esquema vacinal e, consequentemente, na cobertura vacinal, além de aumentar a situação de vulnerabilidade da população em relação aos agravos imunopreveníveis^18^. Assim, estudos de farmacovigilância se mostram muito importantes para melhor compreender o cenário, uma revisão sistemática até o momento demonstra que os EAPV causados por vacinas contra a covid-19 resolveram-se dentro de 24 horas após a vacinação, tendo como reação adversa local mais comum a dor ou sensibilidade na região da aplicação, e como reação adversa sistêmica a fadiga, a febre, ou a dor corporal^19^. Diante do exposto, o objetivo desse estudo foi analisar os EAPV contra o SARS-CoV-2 (COVID-19) no estado de Minas Gerais (MG), Brasil.

## MÉTODOS

Trata-se de um estudo epidemiológico, descritivo, realizado com dados do e-SUS Notifica, no estado de Minas Gerais, durante o período de 20 de janeiro a 5 de março de 2021. Analisou-se todos os casos suspeitos de EAPV da vacina contra covid-19, totalizando 7.305 casos. O fluxograma de seleção da amostra dos EAPV pode ser observado na Figura 1.

**Figura 1:** Casos suspeitos de EAPV-covid-19 notificados no sistema de informação e-SUS notifica, no período de 20 de janeiro a 5 de março de 2021. Minas Gerais, Brasil.

^a^ Taxa de incidência de EAPV, por 100.000 doses aplicadas

O estado de MG é composto por 853 municípios, possui um território de 586.522,122 km^2^e uma população estimada de 21.168.791 habitantes no ano de 2019^20,21^. Para a organização e planejamento da atenção à saúde (em função de suas características demográficas, socioeconômicas, geográficas, sanitárias, epidemiológicas, oferta de serviços e relações entre municípios), o estado é dividido em 14 macrorregiões de saúde: Sul; Centro-Sul; Centro; Jequitinhonha; Oeste; Leste; Sudeste; Norte; Noroeste; Leste do Sul; Nordeste; Triângulo do Sul; Triângulo do Norte e Vale do Aço.

Para este estudo, foram analisados os casos suspeitos de EAPV com e sem encerramento ocorrido no período estabelecido. As variáveis analisadas para os casos de EAPV foram o imunobiológico administrado (AstraZeneca ou Coronavac); o tipo de evento (não grave, grave, erro de imunização ou erro de imunização com evento adverso); a evolução do caso (cura sem sequelas, cura com sequelas, em acompanhamento, não é EAPV, óbito ou outros); e o tempo em dias entre a administração do imunobiológico e o início dos sintomas. Considerou-se também a classificação da causalidade do EAPV (relação temporal consistente, mas sem evidências na literatura para estabelecer uma relação causal, os dados da investigação são: conflitantes em relação à causalidade, inclassificável e associação inconsistente ou coincidente)^15^. Calculou-se também, a Taxa de Incidência (TI) para 100 mil doses aplicadas, utilizando-se a seguinte fórmula:

$$TI=\left(\frac{EAPV}{\text{ Número de doses administradas }}\right)\times 100\text{ mil doses aplicadas }$$

No numerador foi considerado o total de casos de EAPV da vacina de covid-19 e, como denominador, o de doses da mesma vacina administrada no período. O número de doses foi obtido no site da Secretária Estadual de Saúde do Estado de Minas Gerais (SES – MG), informação coletada no dia 5 de março de 2021, dia limite ao qual compreende o período de estudo desta pesquisa, entre 20 de janeiro e 5 de março de 2021.

Para a análise dos dados, foi utilizado o programa *Statistical Software for Professional* (Stata), versão 16.0. As estimativas dos EAPV foram apresentadas em proporções (%), de acordo com o imunobiológico administrado (AstraZeneca ou Coronavac), faixa etária e sexo, além do tipo de evento e evolução do caso. Para o intervalo da administração da vacina e início dos sintomas (em dias), devido à ausência de normalidade da distribuição da variável avaliada pelo teste de Shapiro-Wilk, os dados foram apresentados por meio de mediana.

A pesquisa foi aprovada pelo Comitê de Ética da Universidade Federal de Minas Gerais, sob o protocolo CAAE 53843716.0.0000.5149.

## RESULTADOS

Entre 20 de janeiro e 5 de março de 2021, foram administradas 940.013 doses das vacinas em Minas Gerais, a maior parte como primeira dose (633.032), e 306.981 de segunda aplicação. No mesmo período, foram notificados 7.305 casos de EAPV, o que corresponde a 0,45% do total de doses administradas, com taxa de incidência de 777,12 casos a cada 100 mil doses aplicadas. A maioria dos casos (69%), foi em decorrência do imunobiológico do laboratório AstraZeneca (Figura 1).

Segundo a classificação do tipo do EAPV, 3% foram classificados como EAG (TI: 20,85 EAPV – EAG a cada 100 mil doses aplicadas), sendo que 4,71% desses eventos evoluíram para óbitos (TI: 8,19 óbitos a cada 100 mil doses aplicadas). Contudo, 97% dos casos notificados foram classificados como EANG e 1,11% foram EI (TI: 8,62 EI a cada 100 mil doses aplicadas) (Figura 1).

Sete (0,09) dos casos de EI foram também associados com eventos adversos (EA), com TI de 0,74 a cada 100 mil doses.

Do total de notificações, 43,6% acometeram pessoas com idades entre 18 e 35 anos, incidindo mais sobre as mulheres (83,4%). O tempo entre a vacinação e início dos sintomas teve mediana de seis dias (Tabela 1).

**Tabela 1:** Distribuição dos casos suspeitos de EAPV – covid-19, por faixa etária, sexo e tempo entre vacinação e início dos sintomas. Minas Gerais, Brasil. 2020.

Variáveis	n	%
Faixa etária (anos)		
13 a 17	4	0,1
18 a 35	3.182	43,6
36 a 49	2.736	37,5
50 a 64	969	13,3
≥ 65 anos	398	5,4
Não informado	16	0,2
Sexo		
Masculino	1.204	16,5
Feminino	6.101	83,5
Vacinação e início dos sintomas (dias)^a^	6 (0–44)	

^a^ Mediana (mínimo e máximo).

Em relação aos EAG, a maioria (60,7%) afetou pessoas com 65 anos ou mais e do sexo feminino (65,3%). Os sintomas iniciaram-se oito dias após a administração da vacina (Tabela 2).

**Tabela 2:** Distribuição dos casos suspeitos de EAG – covid-19, por faixa etária, sexo e tempo entre vacinação e início dos sintomas. Minas Gerais, Brasil. 2020.

Variáveis	n	%
Faixa etária (anos)		
18 a 35	25	12,8
36 a 49	28	14,3
50 a 64	24	12,2
≥ 65	119	60,7
Sexo		
Masculino	68	34,7
Feminino	128	65,3
Vacinação e início dos sintomas (dias)^a^	8 (0–35)	

^a^ Mediana (mínimo e máximo).

Foram notificados 77 óbitos e a maioria (89,6%) foi de pessoas com 65 anos ou mais e do sexo feminino (57,1%). O início dos sintomas se deu oito dias após a administração da vacina, sendo que 84,4% foram classificadas como de condições preexistentes, causadas por outros fatores e não pela vacina (Tabela 3).

**Tabela 3:** Distribuição dos óbitos – covid-19, por faixa etária, sexo, tempo entre vacinação e início dos sintomas e classificação da causalidade. Minas Gerais, Brasil. 2020.

Variáveis	n	%
Faixa etária (anos)		
18 a 35	1	1,3
36 a 49	3	3,9
50 a 64	4	5,2
≥ 65	69	89,6
Sexo		
Masculino	33	42,9
Feminino	44	57,1
Vacinação e início dos sintomas (dias)^a^	8 (0 – 35)	
Classificação segundo a causalidade		
Relação temporal consistente, mas sem evidência na literatura para se estabelecer relação causal	1	1,3
Condições preexistentes causadas por outros fatores e não por vacinas	65	84,4
Em investigação	11	14,3

^a^ Mediana (mínimo e máximo).

A maioria dos óbitos era de residentes em instituições de longa permanência para idosos e com comorbidades como adenocarcinoma de próstata, doença pulmonar obstrutiva crônica, neoplasia maligna da próstata, diabetes, insuficiência cardíaca congestiva, hipertensos e doença renal em estágio final. Os diagnósticos de óbito os caracterizaram em septicemia, parada cardíaca, acidente vascular encefálico, infarto agudo do miocárdio, insuficiência cardíaca congestiva, infecção bacteriana, infecção do trato urinário e hipertensão secundária a afecções endócrinas.

Já nos casos notificados de erros de imunização, 43,2% ocorreram a pessoas na faixa etária entre 18 e 35 anos, a maioria mulheres (80,2%) e, do total, sete (8,6%) foram EI com EA (TI: 0,74 a cada 100 mil doses aplicadas).

A classificação dos EI, segundo tipo de ocorrência, pode ser observada na Figura 2. A maioria (27,2%) dos EI foram em decorrência de extravasamento, seguido de vacinação em gestantes (18,5%) fora do grupo prioritário (Figura 2).

**Figura 2:** Classificação dos Erros de Imunização segundo o tipo de ocorrência (n = 81).

## DISCUSSÃO

Os resultados deste estudo demonstram que é frequente a ocorrência dos EAPV em decorrência da vacina contra covid-19 em Minas Gerais. Entretanto, somente 3% foram classificados como graves, com TI de 20,85 a cada 100 mil doses aplicadas, e 4,71% dos EAG evoluíram para óbitos (TI: 8,19 óbitos a cada 100 mil doses aplicadas). Dentre os óbitos analisados, a maioria deles (84,4%) foram classificados como causados por condições preexistentes de outros fatores e não por vacinas, demonstrando que até o momento nenhum dos óbitos apresentou relação causal com as vacinas anticovid-19. Em relação aos EANG, 1,11% foram em decorrência de EI (TI: 8,62 EI a cada 100 mil doses aplicadas)

Os dados deste estudo estão em consonância com os dados publicados no Boletim Epidemiológico do Ministério da Saúde (2021), em que a maioria dos casos notificados foi classificada como EANG e acometeram com maior incidência o sexo feminino. Em relação aos EAG e aos óbitos, a TI para ambos foi inferior à taxa de incidência nacional para o mesmo evento^21^ (TI – EAG: 0,21 *versus* 7,1 por 1.000 doses aplicadas; TI – óbito: 0,08 *versus* 2,4 por 1.000 doses aplicadas, as taxas desses dados foram transformadas para 1.000 doses, para comparação com o Boletim)^22^. Os dados reforçam a segurança e eficácia dos imunobiológicos anticovid-19 disponíveis no Brasil^23,24^.

O dados de perfil sociodemográfico dos casos apresentam maior incidência de EAPV em mulheres com idades entre 18 e 35 anos, resultados que já eram esperados, devido ao fluxo de grupos prioritários seguido pelo esquema vacinal contra a covid-19, iniciando-se com profissionais de saúde^12^, cuja maioria é de profissionais de Enfermagem, em que há predomínio feminino (85,1%)^25^, levando em com que até 5 de março de 2021, 442.099 profissionais de saúde receberam a primeira dose da vacina e outros 246.565 já haviam completado o esquema vacinal contra a covid-19^26^.

A maioria dos óbitos encerrados era de residentes em instituições de longa permanência para idosos (ILPI), pacientes extremamente vulneráveis, debilitados, acamados ou com dificuldades de deambular, além de apresentarem cuidados envolvendo diálise e com múltiplas comorbidades. Os idosos, em especial os residentes de ILPI, também são classificados como grupo prioritário da vacinação contra a covid-19 no Brasil^12^.

O subcomitê de segurança da vacina contra covid-19, do *Global Advisory Committee on Vaccine Safety* (GACVS), em reunião de 19 de janeiro de 2021, analisou relatórios de mortes em idosos classificados como frágeis vacinados com a vacina Pfizer-BioNTech COVID-19 (vacina ainda não utilizada no Brasil durante o estudo) e concluiu que “os relatórios atuais não sugerem qualquer aumento inesperado ou desfavorável de fatalidades em idosos frágeis ou quaisquer características incomuns de eventos adversos, após a administração de Pfizer-BioNTech COVID-19 vacina”^27^.

No Brasil, a Nota informativa Nº 11/2021-CGPNI/DEIDT/SVS/MS, de 2 de fevereiro de 2021, com a finalidade de esclarecer os eventos adversos supostamente atribuíveis à vacinação contra a covid-19, conclui que até o momento, não se pode afirmar tratar-se de EAPV com comprovada associação causal e reforça que, das 37.765 doses aplicadas nos idosos residentes em ILPI, até a data da nota informativa, a incidência de óbitos entre eles foi de 34,4 por 100.000 doses aplicadas, taxa considerada inferior à taxa basal de mortalidade observada nesta população no Brasil (325 a 916 óbitos por mês por 100.000 idosos vivendo em ILPI)^28^.

A ocorrência de erros de imunização foi baixa e, em especial, o número de EI com EA apresentou TI de 0,74 a cada 100 mil doses, que podem ser considerados como qualquer evento evitável que poderá causar ou levar a um uso inapropriado os imunobiológicos ou acarretar dano ao paciente^13^. Os EI podem ser classificados como erro na produção (não cumprimento das boas práticas de fabricação que pode levar a um desvio de qualidade, como alterações de potência e aumento de reatogenicidade); erro na rede de frio (vacina transportada ou armazenada incorretamente); erro no manuseio; e erro na administração (injeção não estéril, erro de reconstituição, injeção no local incorreto, contraindicação ignorada, vacina fora do prazo de validade), que acontecem devido ao não cumprimento de normas e técnicas, podendo resultar em evento adverso^29,30^.

Os EI que resultaram em eventos adversos neste estudo, apresentaram TI menor que 1 a cada 100 mil doses aplicadas. Como eventos evitáveis, é necessário que as coordenações de imunização reforcem os treinamentos sobre a técnica correta de vacinação^31^, em especial as municipais e onde houver a contratação emergencial de vacinadores. Contudo, em campanhas de vacinação em massa, com a imunização contra a covid-19, jé é esperado que ocorram erros de imunização^31^.

Os EI mais comuns foram o extravasamento e a administração em gestante fora do grupo prioritário. Segundo Nota Informativa Nº 21/2021-CGPNI/DEIDT/SVS/MS, de 3 de março de 2021, a ocorrência de extravasamento, seja durante ou após a aplicação, no próprio local da injeção ou na conexão seringa-agulha, deverá ser considerado como EI e, consequentemente, notificada^31^. Entretanto, a Nota reforça que é pouco provável que extravasamentos de pequenos volumes ocasionem pior resposta imune por parte do vacinado e, consequentemente, não se recomenda doses adicionais^31^.

Estudo preliminar de coorte, com número pequeno de participantes gestantes, ao se avaliar soro e leite materno, demonstraram que vacinas de mRNA covid-19 (Pfizer ou Moderna, ainda não vinculadas pelo Ministério da Saúde brasileiro, à época da pesquisa, para vacinar a população), geraram resposta humoral satisfatória em mulheres grávidas e lactantes, com imunogenicidade e reatogenicidade semelhantes às observadas em não gestantes^32^.

A Nota Técnica Nº 1/2021-DAPES/SAPS//MS, de 11 de março de 2021, reforça que até o momento não existem evidências que contraindiquem as vacinas disponíveis no Brasil para as gestantes, puérperas e lactantes e que as pertencentes aos grupos prioritários deverão ser imunizadas^33^. No Brasil, o Grupo Brasileiro de Estudos de covid-19 e Gravidez, identificou que comorbidades são condições associadas significativamente à mortalidade na população de gestantes^34^.

Este estudo, contudo, apresenta algumas limitações, por ser desenvolvida com base em dados de bancos secundários, limitando-se a informações específicas presentes na ficha de notificação, que pode ter inconsistências, uma vez que a inserção das notificações no e-SUS Notifica é realizada por diversos profissionais. Ademais, 14,3 % dos óbitos do período ainda estão em investigação, porém, levando-se em consideração as porcentagens das causas dos óbitos investigados, supôs-se que não haveria alteração na conclusão deste estudo. Todos esses fatores tornam o processo de encerramento e atribuição de causalidade mais lento, lentificando também a análise dos dados produzidos. As Unidades Regionais de Saúde são frequentemente acionadas para que sejam apresentadas tais inconsistências na tentativa de melhorar o processo junto aos municípios notificadores e, assim, melhorar a qualidade dos dados produzidos pelo nível central (área técnica de EAPV de Coordenação de Imunização da Secretaria Estadual de Saúde de Minas Gerais).

## CONCLUSÃO

A maioria dos casos suspeitos de EAPV foram considerados EANG. Esse trabalho fomenta, portanto, a discussão sobre a importância dos registros dos EAPV decorrentes das vacinas contra covid-19, demonstrando que são seguras para a população.

Os resultados encontrados sugerem, ainda, que os óbitos pós-vacina anticovid-19 não estavam relacionados à vacinação, mas foram decorrentes de condições preexistentes causadas por outros fatores, e não pelas vacinas.
